# Conservative Management of an Unusual Congenital Abdominal Wall Defect: A Case Report

**DOI:** 10.7759/cureus.25617

**Published:** 2022-06-02

**Authors:** Ali H Al-Ameer, Ghaida A Alabidi, Yasir G Alrashdan, Abdulwahab Aljubab

**Affiliations:** 1 Pediatric Surgery, King Fahad Medical City, Riyadh, SAU; 2 Pediatric Surgery, King Saud Medical City, Riyadh, SAU

**Keywords:** case report, non-operative, conservative, dysgenesis, abdominal wall defect

## Abstract

Congenital abdominal wall defects comprise a spectrum of anatomical anomalies of the abdominal wall. Most of these anomalies are located in the midline. However, in rare cases, it was lateral and usually found to be a fascial defect with intact skin coverage. We report an unusual case of lateral musculocutaneous abdominal wall defect in a full-term baby boy. The defect is not classified under any of the well-known abdominal wall anomalies. Non-operative management achieved excellent and satisfactory results. Conservative management should always be considered whenever applicable, as it is safe and cost effective.

## Introduction

Congenital abdominal wall defects can be defined as the hypo-development or complete absence of muscular and/or cutaneous layers of the abdominal wall. It includes a wide spectrum of congenital defects. The most common and well-known congenital abdominal wall defects encountered by pediatric surgeons are omphalocele and gastroschisis [1]. Other rarer defects include bladder or cloacal exstrophy and limb-body wall complex. Each of these has a well-established definition and anatomical location [2]. We report a rare abdominal wall defect that is not classified under any of the well-known congenital abdominal wall defects. An extremely small number of similar cases have been reported in the literature.

## Case presentation

A baby boy was delivered at term by normal spontaneous vaginal delivery by a 34-year old mother. She was gravida five, para four, married to a first degree cousin. Both parents are carriers of a mitochondrial mutation (acylglycerol kinase (AGK) gene mutation), a genetic defect that can manifest in Sengers syndrome, a rare autosomal recessive metabolic disorder caused by AGK deficiency [3]. Their first, second, and fourth children died at the ages of one week, nine months, and seven months, respectively, due to complications of Sengers syndrome. None of the siblings had congenital abdominal wall defects. Antenatal history was remarkable for chorionic villus sampling done outside our property at around 14 weeks of gestation. The baby tested negative for the AGK gene mutation. The procedure went smoothly and was uneventful as per the mother. Otherwise, antenatal fetal screening was unremarkable for poly or oligohydramnios, suspicious abdominal wall defect or other congenital anomalies. No history of infections, trauma, smoking, or use of medications was present during the period of pregnancy.

The baby was delivered with an appearance, pulse, grimace, activity, and respiration (APGAR) score of eight and nine at one and five minutes, respectively. He was taken to the neonatal intensive care unit due to mild respiratory distress, the abdominal wall defect, and for a genetic workup. Birth weight was 2,910 grams. On examination, he was a healthy-looking infant with no syndromic features, and his vitals were within normal limits. He was placed on a 2-liter nasal cannula. No cardiac murmurs were appreciated. 

**Figure 1:**
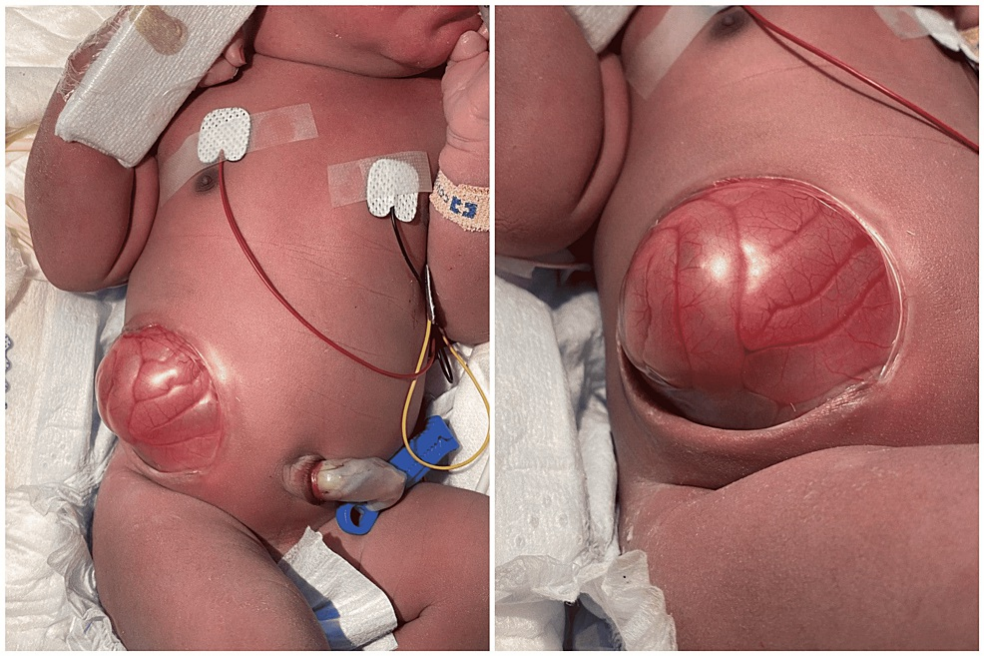
Initial presentation immediately after delivery

**Figure 2:**
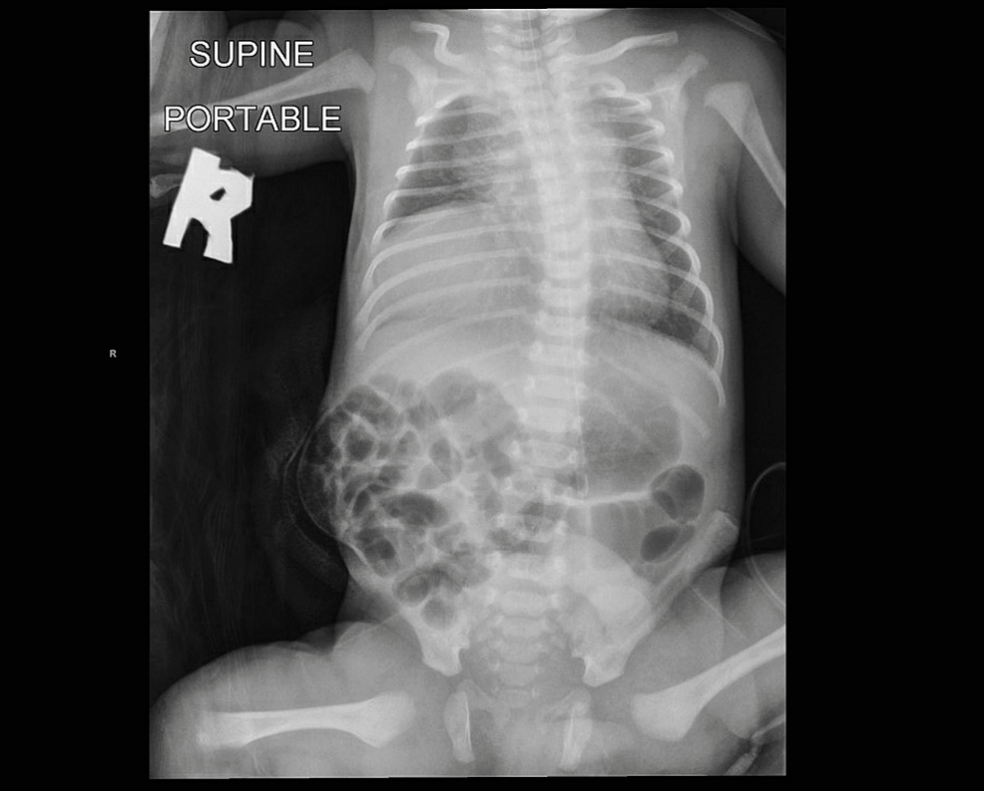
Right-sided diaphragmatic eventration

Abdominal examination revealed normal appearance and position of the umbilical cord. There was a right-lower quadrant abdominal wall defect lateral to the rectus muscles, a “musculocutaneous defect”, round in shape, measuring 6 x 6 cm (Figure 1). The defect was covered with an intact, thin, transparent membrane that bulged higher than the level of the surrounding skin; the small bowel could be seen through the membrane and looked healthy. No liver tissue could be seen. Systemic examination was otherwise unremarkable except for palpable right undescended testis. A babygram was done as part of an evaluation for the mild respiratory distress the baby was having, which showed right sided diaphragmatic eventration (Figure 2).

**Figure 3:**
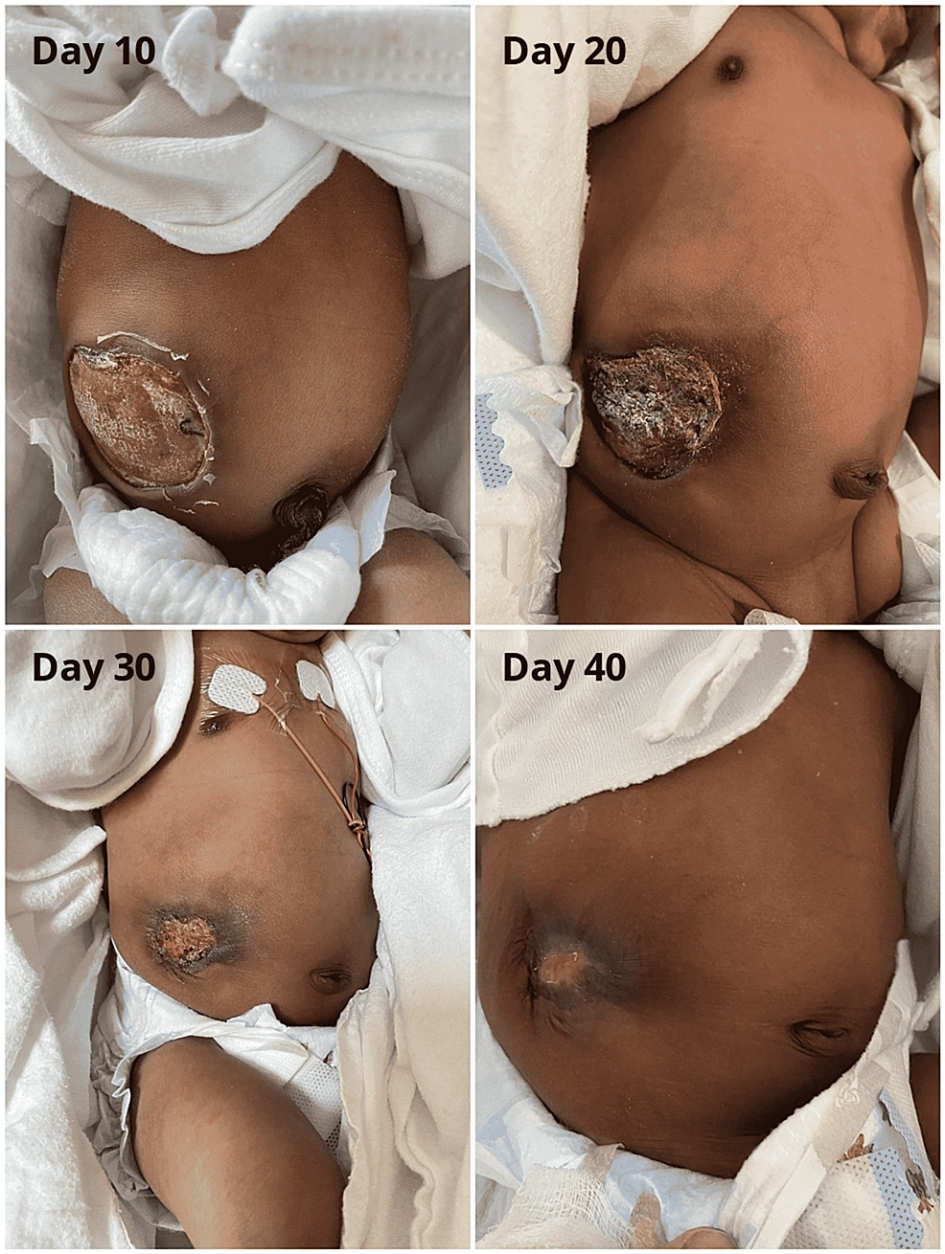
Progression of skin epithelialization over 40 days using the pain and wait approach

We elected to manage the defect conservatively in a similar fashion to what we do in cases of intact omphalocele. Using the paint and wait technique with povidone-iodine, the defect regressed in size until complete skin closure with good epithelization over a period of 40 days (Figure 3). The patient was discharged after complete epithelialization of the defect with clinic follow-up.

**Figure 4:**
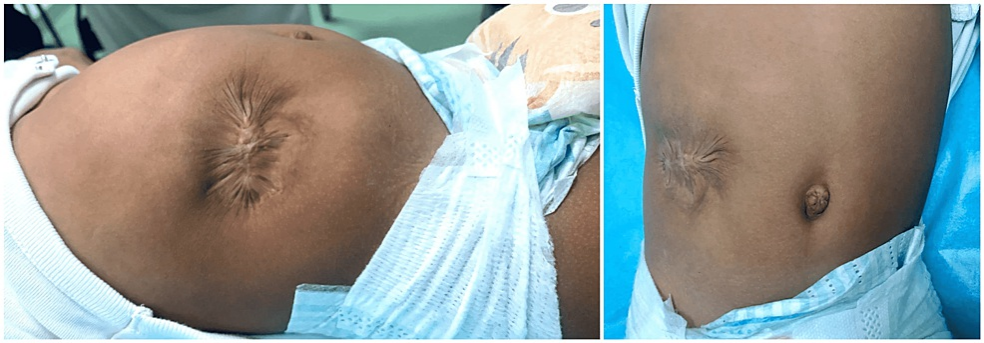
Full skin coverage of the defect, and no fascial defect appreciated on examination at age of 18 months

The baby was routinely followed in the clinic until the age of 18 months. Upon his last visit, the baby was doing well, with no symptoms of respiratory distress or need for hospital visits. The congenital abdominal wall defect was completely covered with healthy skin and no facial defect was appreciated (Figure 4). No bulge was noted even when the baby cried. The patient was booked for elective right orchiopexy.

## Discussion

Different congenital abdominal wall defects are defined by their unique appearance, anatomical location, and associated anomalies. The most commonly encountered defects are gastroschisis and omphalocele. By definition, gastroschisis is a full-thickness abdominal wall defect that is almost always located to the right side of the umbilical cord and has no sac or membrane covering the herniated bowel. In contrast, omphalocele is a mid-line defect that varies in size, with herniated abdominal organs, mostly bowel and possibly liver, being covered by a membrane made of peritoneum, Wharton's jelly, and amnion [1]. In our case, the defect does not fit any of the aforementioned definitions, as it is a lateral defect covered by peritoneum. Arroyo Carrera et al. reported a similar defect and named it a gastroschisis-like defect [2]. We prefer to call it a lateral abdominal wall defect due to its location and presence of membranous bowel coverage.

Abdominal wall defects develop at different stages during embryonic development. Many theories have been proposed to explain the underlying pathophysiology. These are early defects in the germinal disc [4], early rupture of the amnion [5], vascular insufficiency resulting in necrosis of the abdominal wall [6], or abnormality in the embryonic folding process [7]. With regard to the possible association between AGK mutation and this defect, we did not find any association between the two in the literature. However, the baby in our case screened negative to the gene defect. Another possibility was raised regarding the chorionic villus sampling procedure and whether or not it can induce the defect secondary to localized trauma or infection. However, no literature had reported such a complication.

The management options for such lesions are either upfront surgical repair or conservative management. Due to the relatively large size of the defect and concern of tension closure, we elected to manage conservatively using the paint and wait approach since there was intact peritoneal coverage. This approach is well established and routinely used for cases of giant omphalocele where primary closure is not feasible. Simply, we dressed the defect daily with the povidone iodine solution or silver sulfadiazine to induce cicatrization and epithelialization [8]. Patients at this stage can be discharged safely with no concern of sac rupture. Complete spontaneous fascial closure may occur and have been reported in a 6-cm omphaloceles fascial defect [9]. Otherwise, the defect will be managed as a case of ventral hernia.

## Conclusions

Pediatric surgeons may face unusual presentations of abdominal wall defects. No clear explanation for the defect was found in our case. However, invasive intrauterine procedures should be kept in mind as a potential risk factor. Paint and wait approach is safe and feasible option for a sac covered abdominal wall defects. Finally, fascial defect as big as 6 cm can close spontaneously.
